# A BCMA-specific CAR T cell produced with clinically scalable lentiviral and T cell manufacturing processes has potent anti-multiple myeloma activity

**DOI:** 10.1186/2051-1426-3-S2-P124

**Published:** 2015-11-04

**Authors:** Tracy E Garrett, Alena A Chekmasova, John W Evans, Stacie L Seidel, Holly M Horton, Howard J Latimer, Johanna A Griecci, Christopher J Horvath, Byoung Y Ryu, Kevin M Friedman, Richard A Morgan

**Affiliations:** 1 bluebird bio, Cambridge, MA, USA

## 

Chimeric antigen receptors (CARs) operably link T cell activation domains to the antigen recognition properties of an antibody, thereby redirecting T lymphocytes to cells expressing the antigen. B cell maturation antigen (BCMA) is an attractive CAR T cell target to treat patients with multiple myeloma. Nearly all multiple myeloma tumor cells express BCMA, while normal tissue expression is restricted to plasma cells and a subset of mature B cells. We tested four candidate anti-BCMA CARs each comprised of unique single chain variable fragments from antibodies specific to BCMA fused to CD3zeta and CD137 (4-1BB) signaling domains. Candidate anti-BCMA CARs were introduced into T cells via lentiviral vector technology, and the transduced T cells expanded using a scalable clinical manufacturing process. At the end of culture all four candidates demonstrated equivalent transduction efficiencies and released cytokines specifically in response to BCMA-positive multiple myeloma cell lines. One candidate, bb2121, was selected for further studies based on the robust frequency of CAR-positive cells (57.0% +/- 4.5%), and superior *in vitro* cytolytic activity against the multiple myeloma cell line RPMI-8226 after co-incubation at a 10:1 effector to target ratio (77.4%, *P* < 0.0001). To evaluate the anti-tumor activity of bb2121 *in vivo*, we employed a murine xenograft model in which 10^7^ RPMI-8226 cells were subcutaneously implanted in NSG mice eighteen days prior to CAR T cell treatment, resulting in tumor volumes of 95 mm^3^ at the time of infusion. In this setting, a single intravenous treatment of 5.2 x 10^6^ bb2121 anti-BCMA CAR^+^ T cells resulted in rapid tumor infiltration, followed by involution and complete tumor clearance after nineteen days. All mice treated with bb2121 anti-BCMA CAR T cells remained tumor free for the study duration (85 days). In contrast, bortezomib (a current standard of care for multiple myeloma) merely suppressed tumor growth during administration and tumors progressed in all mice by day fifty (n = 10). These data demonstrate the effectiveness of our lentivirus-based CAR T cell manufacturing process and support the further development of bb2121 for the treatment of patients with multiple myeloma.

